# Liquid Phase Exfoliated Hexagonal Boron Nitride/Graphene Heterostructure Based Electrode Toward Asymmetric Supercapacitor Application

**DOI:** 10.3389/fchem.2019.00544

**Published:** 2019-08-02

**Authors:** Xuan Zheng, Guangjin Wang, Fei Huang, Hai Liu, Chunli Gong, Sheng Wen, Yuanqiang Hu, Genwen Zheng, Dongchu Chen

**Affiliations:** ^1^Hubei Provincial Key Laboratory of Green Materials for Light Industry, School of Materials and Chemical Engineering, Hubei University of Technology, Wuhan, China; ^2^School of Materials Science and Energy Engineering, Foshan University, Foshan, China; ^3^College of Chemistry and Materials Science, Hubei Engineering University, Xiaogan, China; ^4^Key Laboratory of Functional Foods, Ministry of Agriculture, Guangdong Key Laboratory of Agricultural Products Processing, Sericultural & Agri-Food Research Institute Guangdong Academy of Agricultural Sciences, Guangzhou, China

**Keywords:** heterostructure, h-BN, graphene, asymmetric supercapacitor, liquid phase exfoliation

## Abstract

In this paper, owing to the electrostatic interaction between graphene and h-BN, a facile liquid phase exfoliation method was carried out to fabricate h-BN/graphene based van der Waals heterostructure nanocomposites without additional chemical cross-linkers. The physicochemical properties of as-prepared composites were characterized by several electron microscopic and spectroscopic measurements. The h-BN/graphene heterostructure composites were employed to use as the anodes of asymmetric supercapacitor, and exhibited exceptional capacitive performance due to their synergistic effects. It is expected that the as-prepared h-BN/graphene materials can boost scalable heterostructure electrodes in supercapacitors, and our liquid phase exfoliation method can be used for the construction of the other energy storage and electronics.

## Introduction

Graphene, as one of the most important representative of 2D nanomaterials, has been a research hotspot in recent years (Kong et al., 2017). It has special Dirac electronic properties (Loan et al., 2014), high carrier migration rate (Morozov et al., 2008), excellent thermal conductivity and mechanical properties (Balandin et al., 2008; Kong et al., 2017), and also been favored by the industry, academia and research institutes. Nevertheless, graphene is a zero band gap material, and its conducting and valence band interlace at the Dirac point. Therefore, the major dilemma to promote the application of graphene in electronic devices is the expansion of band gap. To process with this challenge, researchers have come up with many techniques to expand the bandgap of graphene, including the preparation of graphene nanoribbons (Jiao et al., 2009; Wang and Dai, 2010), nanomesh (Jingwei et al., 2010), and the chemical modification (Li et al., 2011). However, physical etching or chemical reaction would inevitably lead impurities into the boundary or surface of graphene, and greatly reduce the carrier mobility of grapheme (Liao et al., 2014).

Hexagonal Boron Nitride (h-BN) is defined as “white graphene” or “graphene-like” Boron Nitride, with an approximate honeycomb lattice formed by sp2 hybridization and a band gap of 5.9 eV (Wang et al., 2016). Because of its broad band gap, h-BN can be applied in prospects of spintronics, energy storage and composite materials. Because the h-BN and graphene have a rare low mismatched lattice constant, researchers have also revealed new features that the h-BN/graphene heterostructure can regulate the intrinsic electronic structure (Tran et al., 2016). Compared with the single-layer graphene, the carrier mobility (140,000 cm^2^/Vs) of the h-BN/graphene heterostructure film prepared by chemical vapor deposition method is 3.5 times higher than that of the single-layer graphene film (40,000 cm^2^/Vs) (Wang et al., 2013). Besides, the h-BN/graphene heterostructure was proved to have great potential value for application in energy storage devices, like Li-ion battery (Pomerantseva and Gogotsi, 2017; Wu et al., 2017; Li et al., 2018). Researchers also added the h-BN/Graphene heterostructure material into PVA fiber, greatly enhancing the mechanical properties of the composite fiber and increasing the conductivity to 3 S/m, so as to obtain the high-strength conductive composite fiber (Boland et al., 2016). Thus, combining graphene with other 2D materials (such as h-BN) to form heterostructure is bound to greatly expand the research scope of this field like a “snowball.”

At present, large-scale preparation of high-quality h-BN/graphene heterostructure materials is still a recognized problem. From the preparation methods for h-BN/graphene heterostructure, it can be divided into two main modes: one is to grow graphene and h-BN on the substrate surface through CVD (chemical vapor deposition) method, and then transfer and mechanical superposition; the other is to directly use CVD method to grow graphene on substrate and then continue to grow h-BN on the surface of graphene, while this method needs to investigate the lattice mismatch problem. It is noted that the CVD method can ensure integrality of h-BN/graphene heterostructure, but the reaction condition is extremely harsh (usually requires high temperature vacuum environment), the heterostructure size is limited and the cost is high, so it's insufficient to meet the needs of practical application. Therefore, an effective preparation method is urgently needed to make up for the shortcomings of the CVD method to prepare the h-BN/graphene heterostructure materials.

As our best knowledge, liquid phase exfoliation method has been rarely carried out to prepare h-BN/graphene heterostructure. Nevertheless, some investigators have attempted to use this chemical method to create heterostructures, like graphene-black phosphorous-graphene sandwich heterostructure (Sun et al., 2015), metal oxide heterostructures (Xu et al., 2016, 2019; Mahmood et al., 2018; Wan et al., 2018), and (layered double hydroxide) LDH/rGO heterostructure (Ge et al., 2016) which are served as the electrodes in energy storage component. Compared with the heterostructure prepared by CVD method, the liquid phase exfoliation process is simpler and cheaper, and has a broader development prospect.

In this paper, we used the glycerol/urea system in graphene and h-BN exfoliation. The smaller h-BN nanosheets were attached on bigger graphene through ultrasonic assistant. Based on a certain amount of space size differences, the phenomenon of reunification in h-BN or graphene can be effectively prevented under the action of electrostatic force. Furthermore, the h-BN/graphene heterostructure system prepared in this work can be stably dispersed in organic solution, and the system can maintain long-term stability. Moreover, the h-BN/graphene heterostructure materials with different mass ratios in application of supercapacitors is studied in detail. As a result, the h-BN/graphene heterostructure materials show the maximum capacitance of 134 F/g and good cycling stability (96 % of the initial capacitance after 10,000 cycles at 10 A/g). Meanwhile, the assembled asymmertic supercapacitor (ASc) exhibits maximum energy density of 2.05 Wh/kg at high power density of 1998.5 W/kg.

## Experimental Section

### Materials Preparation

Graphite powder (300 mesh, purity>95%, XFNANO Materials) or h-BN powder (1 um, purity>98%, Sigma-Aldrich) were dissolved in the urea/glycerol (molar ratio = 2:1) dispersion. After that, 200 mL of the graphite or h-BN dispersion were transferred to a 800 mL flat bottom beaker, under which graphite or h-BN powder were exfoliated and dispersed through mechanical stirring at 800 rpm for 24 h. Then, the obtained products were evenly transferred to a 50 ml centrifuge tube, then centrifuged for 25 min at 5,000 rpm. The top half of the centrifuged graphite or h-BN dispersion were collected and re-dispersed in DMF, followed by filtration and ultrasonic washing with large amounts of DMF and ethanol, and drying in vacuum oven at 60°C. The yield of graphene or h-BN was decided by taking off the mass of the residual solid (Zheng et al., 2018).

The graphene/DMF and h-BN/DMF dispersion solution were mixed together with a certain mass ratio (the mass content ratio of graphene and h-BN was 1:2, 1:1, 2:1, respectively). The resulting mixed solution was ultrasonic for 30 min, then stirred at room temperature for 24 h and centrifuged at 1,000 rpm for 30 min. The upper liquid was discarded, and the solid precipitation was finally obtained, namely, h-BN/grapheme (BN/G) heterostructure materials.

### Characterization

Transmission electron microscopy (TEM) and high resolution transmission electron microscopy (HRTEM) images were obtained using a JEM-2001F (JEOL, 200 kV primary beam) equipped with a Gatan CCD camera. The morphology of the samples was observed by using S-4800 (Hitachi, accelerating voltage of 25 KV) cold field emission scanning electron microscope (FESEM). The crystal structure of heterostructure was analyzed by D8 Advance X-ray diffraction systems (Bruker, λ = 0.154056 nm). Raman spectra of the samples were tested by an InVia (Renishaw, UK) spectrometer to reflect the composition equipped by a 633 nm laser. The thickness of all samples was also analyzed by MFP-3D-SA AFM (AsylumResearch, USA) using a tapping mode at a scan rate of 1 Hz.

### Electrode Preparation and Electrochemical Tests

The BN/G working electrodes were prepared by mixing 90 wt.% active material (BN/G heterostructure materials) and 10 wt.% polyvinylidene fluoride (PVDF) in NMP solvent to form a slurry. Then the slurry was coated onto a clean Ni mesh (current collector) and dried in a vacuum oven at 60 °C for 24 h. The counter electrode was the activated carbon (AC, SSA = 1,800(±100) m^2^/g, purity>94.2%, XFNANO Materials). The counter electrodes were prepared via the same method as the above working electrodes. The loading mass of each electrode was about 0.15 mg/cm^2^. Microporous polypropylene and 2 M KOH solution were used as the separator and electrolyte, respectively.

The electrochemical performance of samples was measured on the CHI660E (Chenhua, China) electrochemical workstation. Cyclic voltammetry (CV) tests were carried out at the scan rate of 10~200 mV/s. Galvanostatic charge-discharge (GCD) tests were performed at various current densities from 0.5 to 10 A/g. Electrochemical impedance spectroscopy (EIS) was employed in a frequency from 0.01 Hz to 100 kHz at an amplitude of 5 mV. All the related electrochemical tests were performed at room temperature. Specific capacity of the BN/G in two-electrode was calculated from Equations (1) and (2) (Wang et al., 2014):

(1) $$C=\frac{\int I_{1}dV}{\text{sm}\Delta \text{V}}$$

(2) $$C=\frac{{\text{I}}_{2}\text{t}}{\text{m}\Delta \text{V}}$$

Where *I* represents the discharged current (A), s is the scanning rate (V/s), Δ*t* and Δ*V* are the discharged time (s) and the voltage drop upon discharging, respectively, and m is the mass of the electroactive materials (g). The energy density (*E*, Wh/kg) and the power density (*P*, W/kg) of the BN/G//AC were calculated based on the following Equations (3) and (4) (Balogun et al., 2016):

(3) $$E=0.5CV^{2}/3.6$$

(4) $$P=\frac{E\times 3600}{t}$$

Where *C* represents specific capacity of the capacitor (F/g) calculated according to Equation (2), Δ*t* and Δ*V* are the discharged time (s) and the voltage drop upon discharging, respectively.

## Results and Discussion

**Figure 1:**
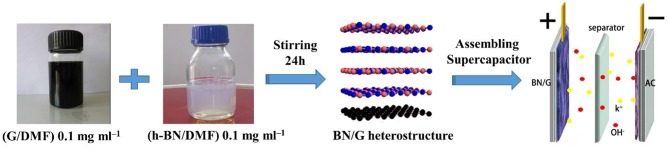
Schematic illustration for the preparation of the BN/G heterostructure.

**Figure 2:**
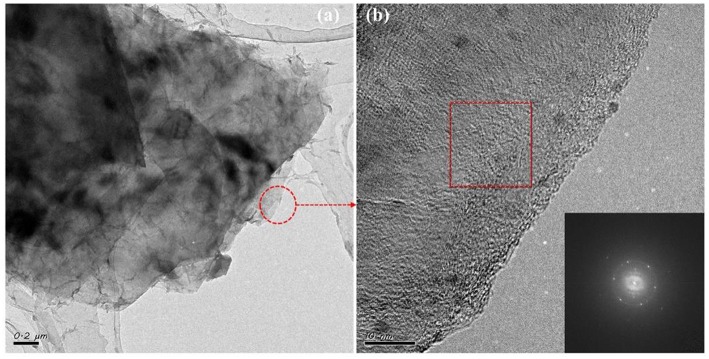
The TEM **(a)** and HRTEM **(b)** images of graphene, the inset is fast fourier transform (FFT) image.

Figure 1 illustrates the preparation of the BN/G heterostructure. The graphene/DMF and h-BN/DMF dilute solution were added into the flat bottom beaker, respectively. Then the mixture was stirred to force the exfoliated h-BN nanosheets entering into the graphene layers to form heterostructure through π-π accumulation. Firstly, the morphology and structure of as-prepared graphene were characterized to verify the effectiveness of glycerol/urea liquid phase exfoliation system. In this work, due to the effectiveness of the exfoliation system, graphene/DMF dispersion solution was randomly diluted to a certain concentration to observe its micromorphology through TEM. As shown in Figure 2a, the graphene exhibited a dispersive lamellar distribution under a low magnification, whilst the unique wavy lattice fringes of graphene were further observed in the high magnification (Figure 2b). and these small lattice fringe is peculiar to the crystal of graphene material. At the same time, dotted impurities can be observed, which is most likely due to the non-covalently modified π-π accumulation effect formed by the interaction of urea or solvent with graphene in the glycerol/urea system (Chen et al., 2018). The selective fast fourier transform (FFT) analysis of the partial amplification of graphene in Figure 2b revealed the unique hexagonal pattern of electron diffraction in the few-layer of graphene. This result can clearly show the successful exfoliation of graphite (Paton et al., 2014).

In order to further observe the graphene prepared by the glycerol/urea system mentioned above, the graphene/DMF dispersion samples were analyzed with the SEM morphology. Figure S1a was the dispersion diagram of graphene after exfoliation, comparative measurements on scale bar of 1 um suggested that the size of graphene was close to 6 um. In addition, according to the morphology at high magnification (Figure S1b), the edges of graphene sheets were corrugated, and there were obvious upwarp, verifing the successful exfoliation of graphite.

We also used AFM to conduct random sample analysis of the exfoliated graphene, which was compared with the sampling method of TEM: the sample to be tested was dripped with pipette gun to the newly prepared mica film after vacuum dust removal. It can be observed the 2D morphology of typical graphene in the tapping mode from Figure S2. The size of the graphene lamella was also more than 5 um, which as similar with the observed size of TEM and SEM. Based on the theoretical thickness of graphene (0.34 nm), the thickness of two adjacent areas in view of Figure S2 is about 0.68 nm (Pattammattel and Kumar, 2015). This value indicated that the as-prepared graphene was close to double-layer, showing the exfoliated graphene by glycerol/urea system was few-layer.

**Figure 3:**
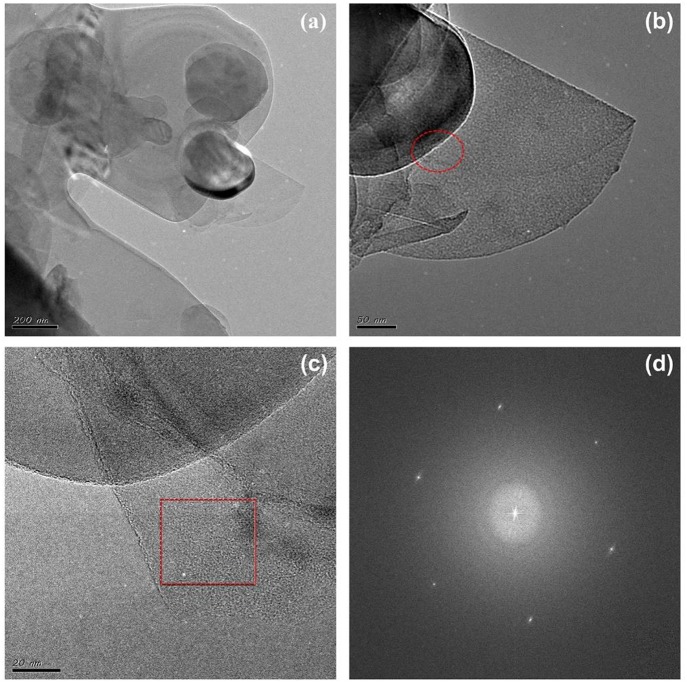
The TEM **(a–c)** and FFT **(d)** images of h-BN nanosheets.

To test the universality of glycerol/urea in the above system, we used the same process to treat other 2D material, such as h-BN, and also obtained h-BN/DMF dispersion solution. Then the h-BN samples were observed by TEM. As shown in Figure 3, it can be seen that the h-BN nanosheets were stacked on top of each other, and their sizes were below 1 um, much smaller than the size of graphene. From the HRTEM images (Figures 3b,c), it can be preliminarily concluded that h-BN powders were exfoliated into nanosheets. Combined with the FFT image in Figure 3d, the obtained h-BN lamination belongs to few-layer, which also proves the availability of glycerol/urea system (Varrla et al., 2015).

Figure S3 is the SEM images of h-BN nanosheets. The white hexagonal circular lamella in Figure S3b is h-BN nanosheet with a size of <1 um, which is consistent with the intuitive size of TEM photographs. Figure S3c shows that h-BN nanosheet have obvious vertical stratification, indicating that h-BN raw powder is peeled into h-BN nanosheet. Figure S4 shows the AFM diagram of h-BN sample, the thickness of the two adjacent white crystals is about 0.7 nm, which is in line with the theoretical value of the double-layer h-BN nanosheet (Kim et al., 2011; Tran et al., 2016).

To understand the crystal structure characteristics of the as-prepared BN/G heterostructure samples, XRD analysis was performed in Figure S5. It can be concluded that the graphene has extremely sharp diffraction peak at 26.5°, and h-BN has obvious diffraction peak at 26.7°, which is mainly reflected by the relatively small layer spacing difference between h-BN and graphene. The (002) crystal faces of graphene and h-BN still show no obvious deviation, implying that the formation of heterostructure is only a physical stacking process.

**Figure 4:**
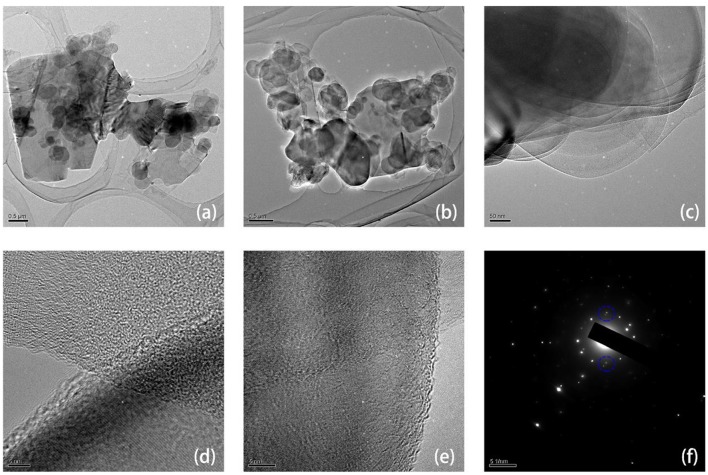
The TEM **(a–e)** and selected area electron diffraction (SAED) **(f)** images of h-BN/graphene heterostructure.

Figure S6 shows the typical Raman absorption peaks of h-BN/graphene, h-BN and graphene, such as D peak (~1,331 cm^−1^) and G peak (~1,578 cm^−1^), which are shown in Graphene and BN/G, respectively, demonstrating the presence of graphene in BN/G heterostructure. Due to the strong photoluminescence background of h-BN, the photoluminescence background shown in the BN/G region can be attributed to the defect states of h-BN, including defects along grain boundaries (Li et al., 2012). The heterostructure of BN/G was determined by HRTEM and SAED. Figures 4a,b depicted two BN/G heterostructure regions, in which the larger graphene sheet was covered by the smaller h-BN nanosheets. This structure size is in accordance with the previous TEM results of h-BN and graphene. The HRTEM image in Figures 4c–e further proved a certain difference between exfoliated h-BN and graphene on the interface edge of the BN/G heterostructure, and the lattice fringe of the two structures was particularly obvious. Figure 4f is the SAED result of lattice fringe corresponding to BN/G heterostructure. From its diffraction pattern, a set of clear hexagonal diffraction spots can be observed. The close view of the area indicated by the blue circle shows two separate diffraction points along the radial direction. These two points correspond to (100) plane diffraction of h-BN and graphene, respectively. The calculated plane spacing of the two points was 2.13 and 2.06 Å, which were in good agreement with the (100) crystal plane spacing values obtained by the XRD tests. The AFM height of BN/G heterostructure on mica substrate is shown in Figure S7. The h-BN is small in size and stacked on top of the graphene layers in different thicknesses. However, the average thickness of graphene is larger than that of the previous graphene sheet, attributing to the alternating superposition of h-BN and graphene in the heterostructural materials (Yang et al., 2013a).

Inhibiting the stack of graphene is the key factor to realize high the performance of the electrochemical capacitor based on graphene materials. As for rGO, the capacitance is significantly reduced when re-stacking between graphene sheets, mainly due to the irreversible stacking of single rGO sheet during the reduction and drying process (Yang et al., 2013b). We expect that the h-BN nanosheet in the BN/G can be used as an effective electrolyte channel to increase the overall electroactive surface area (Figure 1). It is hoped that charges can be stored in graphene or h-BN electrodes through electrostatic interaction, adsorption and desorption of interface ions (Figure S8). Since graphene can be assumed as a zero-gap semiconductor with Fermi energy level located at Dirac point, the presence of B and N in h-BN makes it form h-BN/graphene superlattice above the Fermi energy level (Ge et al., 2016; Lee et al., 2016). Therefore, we prepared BN/G with different mass loads of h-BN and studied the potential application of h-BN as a 2D structural carrier and electrolyte channel in graphene-based membrane.

**Figure 5:**
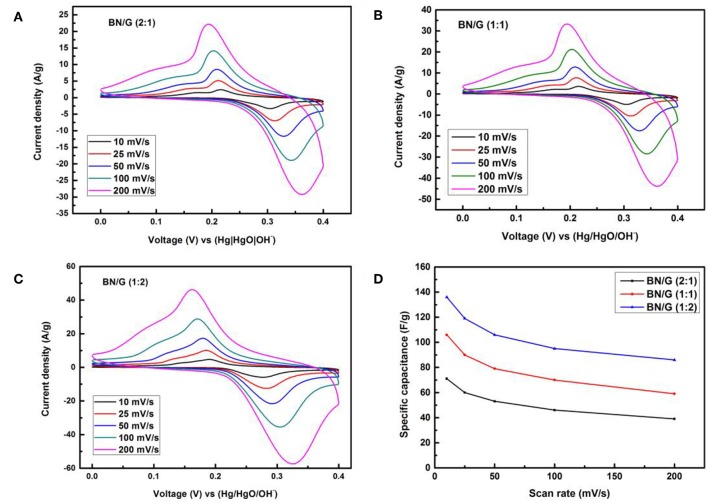
CVs of all BN/G at different scan rates **(A–C)** and **(D)** tendency of the specific capacitance of three BN/G samples at scan rates of 10–200 mV/s.

Figures 5A–C show the CV curves of three mass ratios BN/G measured by the two-electrode method at different scanning rates (10 ~200 mV/s) in 2 M KOH. As illustrated in Figures 5A–C, we can notice the prominent redox peak, which ascribing to the change of oxidation state of N atoms by electrolyte insertion. Figure 5D is the change of capacitance with the increase of scan rates, the CV curve still shows a larger redox peak even when scan rate increases to 200 mV/s, this is because the Fermi level of BN/G electrode materials reached a higher potential compared to the redox potential of electrolyte, electrons transferred from the electrode to the interface of the electrolyte. Since h-BN is usually used as dielectric material, its capacitance is very low, while the capacitance of BN/G is relatively large, it possibly attributed to BN/G heterostructure shortening the ion transport path and increasing the specific surface area for charge storage.

**Figure 6:**
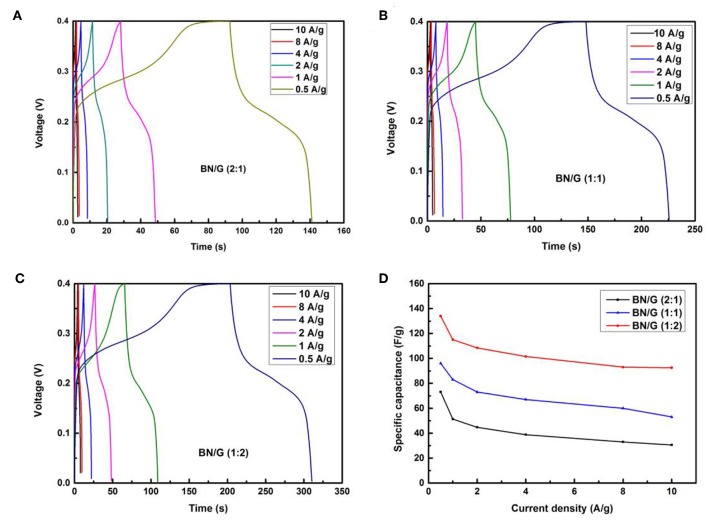
Galvanostatic charge–discharge curves for all BN/G at different current densities: **(A)** BN/G(2:1), **(B)** BN/G(1:1), **(C)** BN/G(1:2), and **(D)** specific capacitance of all BN/G as a function of discharge current.

However, due to the differences in the contributions of h-BN and graphene to the overall capacitance in BN/G, the accumulation of h-BN multilayer and the increase of Faraday resistance may be the reason for the lower capacitance of BN/G(2:1; 1:1). It is found that the graphene is inserted into the interlayer space of h-BN to play the role of interval layering, preventing the re-stacking of h-BN nanosheets. Therefore, the charge storage mechanism in BN/G may be caused by the synergistic effect of graphene and h-BN. Figures 6A–C shows the charge and discharge curves of BN/G samples with different mass ratios at different current densities. The specific capacitance value is calculated according to the above Equation (2). Compared with BN/G(2:1) and BN/G(1:1), the discharge time of BN:G(1:2) is longer at the same current density. Furthermore, BN:G(1:2) has a specific capacitance of 134 F/g when the current density is 0.5 A/g. By contrast, BN/G(2:1) and BN/G(1:1) have specific capacitance of 71 and 106 F/g, respectively, at the same current density.

The variation trend of BN/G samples with different mass ratios is shown in Figure 6D. Driven by the increase of current density, the capacitance of BN/G declined, but it tended to be flat at current densities ranged from 2 to 10 A/g, indicating the BN/G heterostructural materials have good rate performance. When the current density is increased to 10 A/g, the capacitance retentions of BN/G(2:1), BN/G(1:1), and BN/G(1:2) are 55, 56, and 69 %, respectively. These capacitance retentions match well with the trend calculated by the CV method in Figure 5D, and the optimal ratio is BN:G = 1:2.

**Figure 7:**
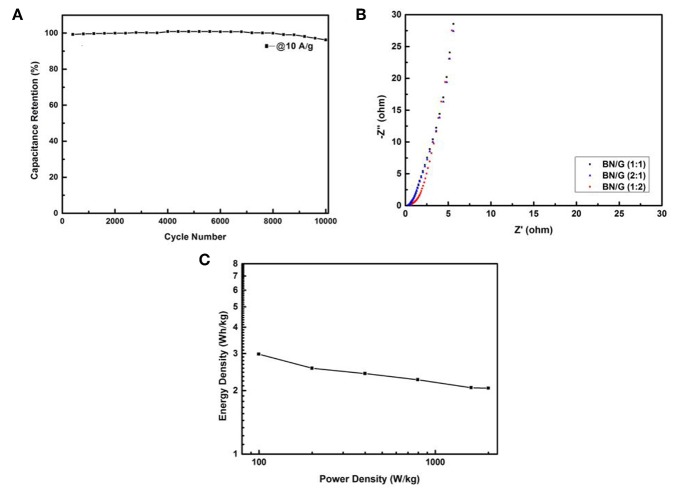
Cyclic performances of the BN/G (1:2) after 10,000 cycles at 10 A/g **(A)**, Nyquist plots of all BN/G **(B)**, Ragone plots of the BN/G (1:2) **(C)**.

BN:G(1:2) was further taken as a representative to study its cycle stability for charging and discharging 10,000 times under the current density of 10 A/g. Figure 7A shows that after 10,000 times of rapid charge and discharge tests, BN:G(1:2) electrode has only 4% loss, exhibiting its excellent electrochemical stability. This is because h-BN has extremely high chemical and thermal stability, providing synergies with high electrochemical stability when it exists in conductive graphene systems (Kumar et al., 2015). Figure 7B shows the AC impedance diagram of BN/G samples with different mass ratios. The lattice in the figure shows that the BN/G sample exhibits excellent capacitive characteristics, especially the approximate vertical line in the lower frequency range. And the charge transfer resistances (Rct) of BN/G(2:1), BN/G(1:1), and BN/G(2:1) are small, and the equivalent series resistance (ESRs) is 6.7, 6.0, and 5.3 Ω, respectively. The results can be concluded that h-BN in heterostructure creates electrolyte channel, and as an effective ion channel to boost and expedite diffusion of electrolyte ions such as K^+^ and OH^−^ (Hu et al., 2014).

Toward studying the electrochemical properties of our assembled BN/G//AC ASc, we further reflected its power density and energy density. Figure 7C showed the power-energy density relation curve of BN/G(1:2). The calculated values of the energy density and power density of BN/G(1:2) were obtained according to Equations (3) and (4). It can be seen from Figure 7C that the energy density of BN/G(1:2)//AC attenuates very little with the increase of power density. Even at the power density of 1998.5 W/kg, its energy density still retains at 2.05 Wh/kg, showing the bright prospects for the application of h-BN/grahene-based supercapacitors.

## Conclusions

In summary, we reported a well-constructed heterostructure materials, in which the h-BN nanosheets were assembled with graphene through electrostatic interaction based on solution method. This scalable stacked heterostructure are well-aligned in vertical, but the layers are randomly stacked in horizontal. The synthesis method adopted in this work has extensive use, low cost and facile process, which can be applied to other 2D materials. Meanwhile, we further developed BN/G heterogstructure electrode material, and explored the feasibility of BN/G heterostructure electrode material in the application of asymmertic supercapacitors. The incorporation of h-BN not only constructs the electrolyte channel for the graphene layer, but also improves the electrochemical performance. Moreover, the assembled BN/G//AC ASc exhibits maximum energy density of 2.05 Wh/kg at high power density of 1998.5 W/kg, and excellent long cycling stability with 96% of initial specific capacitance after 10,000 cycles at 10 A/g, indicating its promising application in energy storage component.

## Data Availability

The raw data supporting the conclusions of this manuscript will be made available by the authors, without undue reservation, to any qualified researcher.

## Author Contributions

XZ, GW, FH, HL, and CG were responsible for all the experiments and the analysis of data. FH, SW, YH, GZ, and DC were responsible for the drafting. All authors contributed equally to the final writing of the paper.

### Conflict of Interest Statement

The authors declare that the research was conducted in the absence of any commercial or financial relationships that could be construed as a potential conflict of interest.
