# Supplementary material for: Liquid Phase Exfoliated Hexagonal Boron Nitride/Graphene Heterostructure Based Electrode Toward Asymmetric Supercapacitor Application
Source: Front Chem. 2019 Aug 2;7:544. doi: 10.3389/fchem.2019.00544 (PMC6688068; doi:10.3389/fchem.2019.00544)
Supplement: Supplementary file 1 [file Data_Sheet_1.PDF]

Supplementary Material for

Hexagonal boron nitride/graphene heterostructures for asymmetric supercapacitor

Xuan Zheng [1,2], Guangjin Wang [1,2,*], Fei Huang [3], Hai Liu [2], Chunli Gong [2], Sheng Wen [2], Yuanqiang Hu [2], Genwen Zheng [2], Dongchu Chen [1,*]

1 School of Materials Science and Energy Engineering, Foshan University, Foshan 528000, China

2 College of Chemistry and Materials Science, Hubei Engineering University, Xiaogan 432000, China

3 Sericultural & Agri-food Research Institute Guangdong Academy of Agricultural Sciences, Key Laboratory of Functional Foods,Ministry of Agriculture, Guangdong Key Laboratory of Agricultural Products Processing, Guangzhou 510610, PR China

* Corresponding author: wgj501@163.com (G-J Wang), Chendc@fosu.edu.cn (D-C Chen)

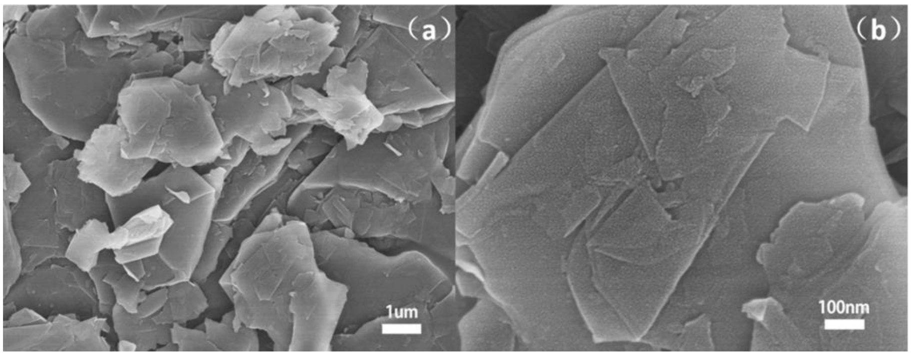

Fig. s1 The SEM images of graphene.

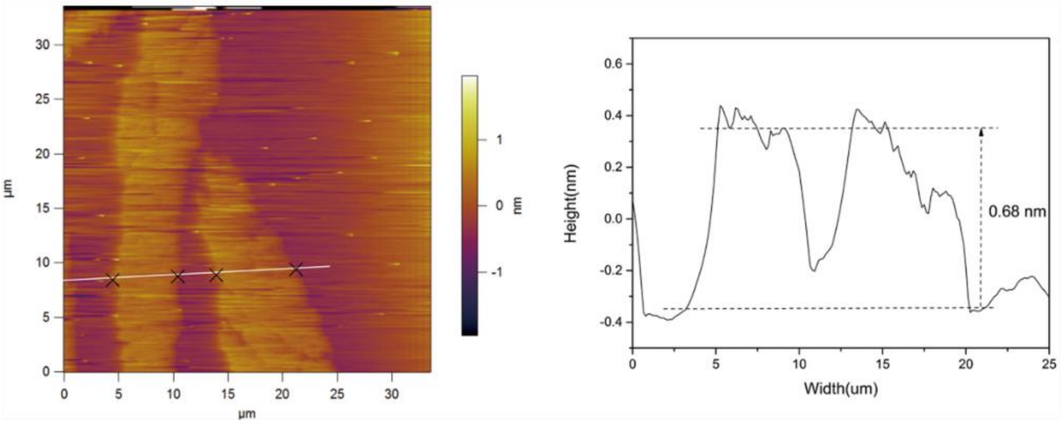

Fig. s2 The atomic force microscopy (AFM) elevation of graphene.

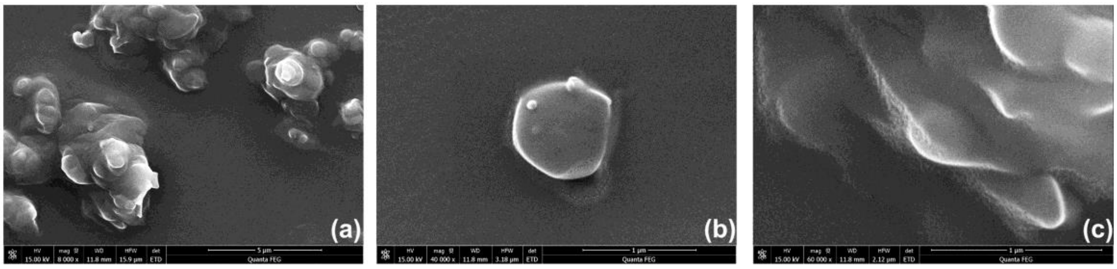

Fig. s3 The SEM (a-c) images of h-BN nanosheets.

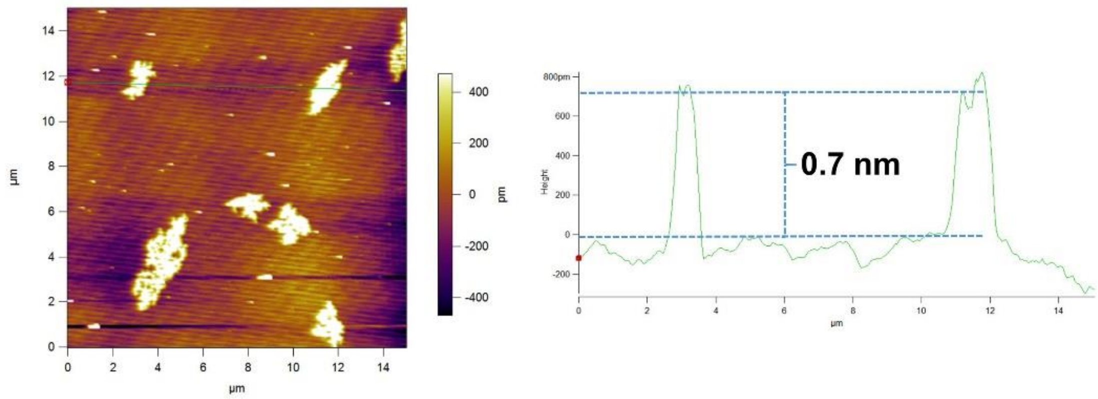

Fig. s4 The atomic force microscopy (AFM) elevation of h-BN nanosheets.

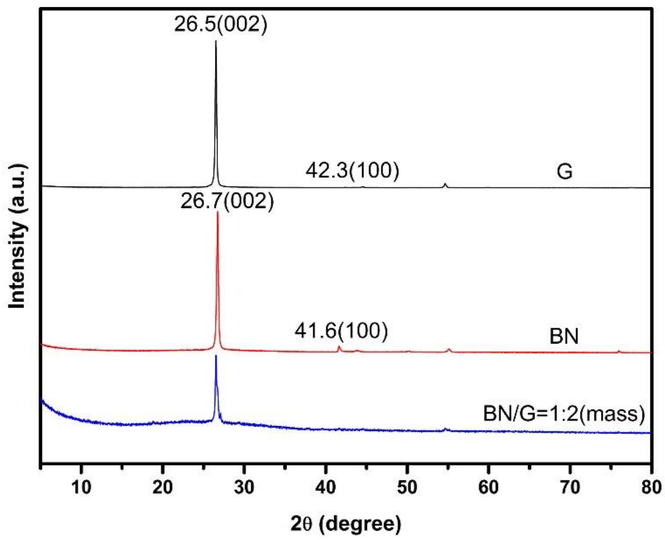

Fig. s5 The XRD patterns of graphene, h-BN nanosheets and h-BN/graphene heterostructure.

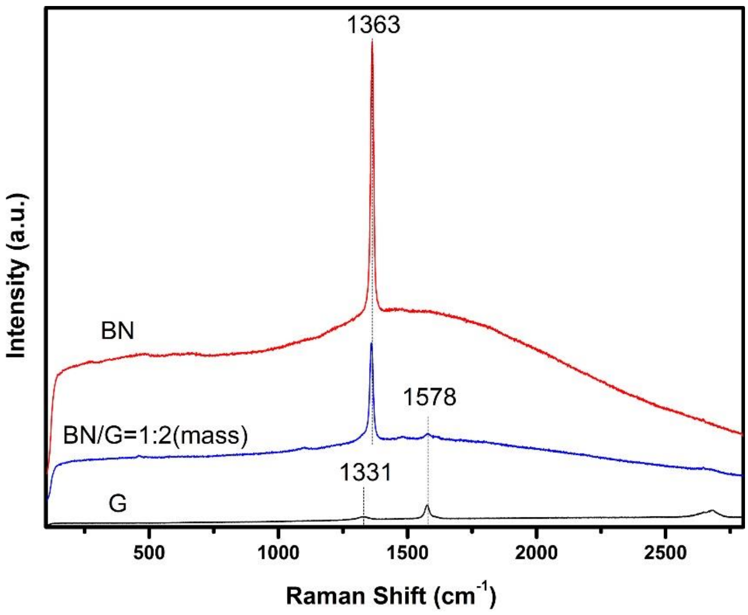

Fig. s6 Raman spectra of graphene, h-BN nanosheets and h-BN/graphene heterostructure.

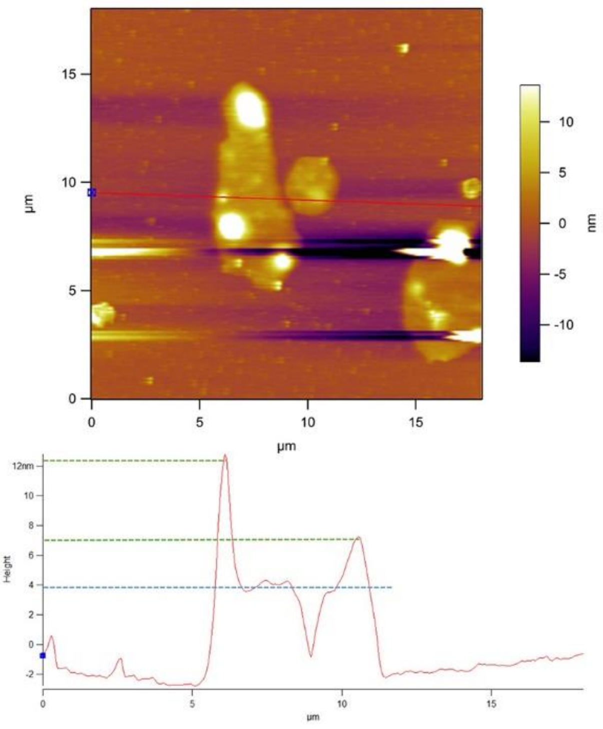

Fig. s7 The atomic force microscopy (AFM) elevation of h-BN/graphene heterostructure.

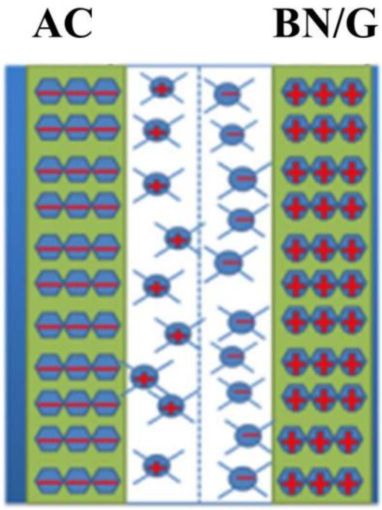

Fig. s8 Schematic illustration of the BN/G//AC ASc.
